# Epstein–Barr Virus in Inborn Immunodeficiency—More Than Infection

**DOI:** 10.3390/cancers13194752

**Published:** 2021-09-23

**Authors:** Ciro Novaes Rosa Lino, Sujal Ghosh

**Affiliations:** Department of Pediatric Oncology, Hematology and Clinical Immunology, Center of Child and Adolescent Health, Medical Faculty, Heinrich-Heine-University, 40225 Duesseldorf, Germany; ciro.novaesrosalino@med.uni-duesseldorf.de

**Keywords:** Epstein–Barr Virus, EBV, inborn errors of immunity, cancer, lymphoma, immunodeficiency

## Abstract

**Simple Summary:**

Epstein–Barr Virus (EBV) is a common virus that is readily controlled by a healthy immune system and rarely causes serious problems in infected people. However, patients with certain genetic defects of their immune system might have difficulties controlling EBV and often develop severe and life-threatening conditions, such as severe inflammation and malignancies. In this review, we provide a summary of inherited immune diseases that lead to a high susceptibility to EBV infection and discuss how this infection is associated with cancer development.

**Abstract:**

Epstein–Barr Virus (EBV) is a ubiquitous virus affecting more than 90% of the world’s population. Upon infection, it establishes latency in B cells. It is a rather benign virus for immune-competent individuals, in whom infections usually go unnoticed. Nevertheless, EBV has been extensively associated with tumorigenesis. Patients suffering from certain inborn errors of immunity are at high risk of developing malignancies, while infection in the majority of immune-competent individuals does not seem to lead to immune dysregulation. Herein, we discuss how inborn mutations in *TNFRSF9*, *CD27*, *CD70*, *CORO1A*, *CTPS1, ITK*, *MAGT1*, *RASGRP1, STK4*, *CARMIL2, SH2D1A*, and *XIAP* affect the development, differentiation, and function of key factors involved in the immunity against EBV, leading to increased susceptibility to lymphoproliferative disease and lymphoma.

## 1. Introduction

Epstein–Barr Virus (EBV) is a gammaherpesvirus with a prevalence of over 90% in the adult population. In immune-competent patients, EBV establishes a life-long latent infection [1,2,3]. Most individuals are infected during childhood with few or no overt symptoms. Adolescents and young adults usually develop infectious mononucleosis (IM), a self-limiting illness with fever, sore throat, lymphadenopathy, hepatosplenomegaly, and fatigue, caused by acute inflammation and hyperactivation of CD8^+^ T cells [4,5].

Primary EBV infection occurs mainly through the oropharyngeal epithelium transmitted by saliva [6]. The lytic infection of the epithelium is followed by a high tropism of the virus towards B cells in which it switches to its latent program. Naïve B cells are driven by EBV into full latency (stage III, during which all latency genes are expressed Epstein–Barr nuclear antigen (EBNA)-1, 2, 3A, 3B, 3C, and LP, Latent membrane protein (LMP)-1, 2A and 2B, EBV-encoded small RNAs (EBERs), and Bam-HI A rightward transcripts (BARTs)). During further progression, EBV gradually reduces the number of encoded genes. Naïve B cells migrate to the germinal center and undergo further expansion. At the germinal center stage, B cells show a restricted gene expression profile (EBNA-1, LMP-1, 2A and 2B, EBERs, and BARTs), known as latency II, mediating survival and differentiation of EBV-infected B cells into memory cells. Finally, EBV-infected memory B cells, the site of virus persistence, further restrict their expression program to EBERs and BARTs only (latency 0), or additionally EBNA-1 (latency I) during homeostatic proliferation.

Occasionally, EBV turns to its lytic program in plasma cells, leading to the production of new virions, repeated epithelial infection, and shedding of viral particles into the saliva [7,8,9,10]. Viral antigens expressed by EBV during its lytic and latent stages are highly immunogenic and induce a strong response against infected cells. Hence, the downregulation of such molecules is essential to escape immune surveillance and provide virus persistence [11,12,13].

Natural killer (NK) and T cells play a major role in controlling EBV. Viral infection decreases MHC class I expression, but natural killer (NK) cells can recognize this state and destroy the cells. EBV lytic infection causes suppression of MHC class I expression and induction of expression of CD112 and UL16 binding protein 1, NK cell activation receptor ligands [14]. Thus, lytic infected cells are eliminated by NK cells, but most EBV infections evade NK cell attack by shifting to latent infection [14,15,16]. In humanized mice, which were challenged with EBV, depletion of NK cells caused exacerbated IM symptoms, with higher viral loads, larger spleens, increased weight loss, and more tumor burden [15].

Cytotoxic CD8^+^ T cell responses play an even bigger part in the immune response to EBV, addressing both lytic and latent stages of infection [17]. During IM, EBV-specific CD8^+^ T cells targeting mainly lytic proteins can expand up to 50% of the circulating CD8^+^ T cell pool. [18,19]. CD4^+^ T cells recognize a variety of EBV epitopes; however, their expansion is much less [20,21]. Interestingly, some CD4^+^ T cells develop a cytotoxic phenotype, with expression of granzyme and perforin, and are able to lyse lymphoblastoid cell lines (LCLs) and EBV loaded peripheral blood mononuclear cells (PBMCs) [22,23,24]. Recently, the impact of γδ and natural killer T (NKT) cells on immunity against EBV could be partially delineated. A comprehensive review of the T cell response to EBV, including unconventional populations, was conducted by Long et al. [25].

The importance of T cells to control EBV can be observed in several conditions in which effector cells are compromised, such as aging, human immunodeficiency virus (HIV) infection, transplantation, or as reviewed here, inborn errors. In those individuals, persistent reactivation and proliferation of EBV-infected cells are associated with severe pathologies that can have lethal outcomes [26,27,28,29]. In this review, we will discuss genetic diseases, which lead to uncontrolled EBV-associated immune dysregulation.

## 2. Inborn Errors of Immunity (IEI)

IEI (also known as primary immunodeficiencies) are a heterogeneous group of diseases, in which patients manifest with increased susceptibility to infections or other immunological disturbances such as autoimmunity, autoinflammation, or immune dysregulation [30]. These conditions result from germline mutations affecting the development, differentiation, and/or function of the immune system. More than 430 genes have been associated with specific diseases; due to next-generation sequencing technologies, this number is constantly growing [30]. IEI are expected to affect 1/1000 to 1/5000 births [31].

**Figure 1:**
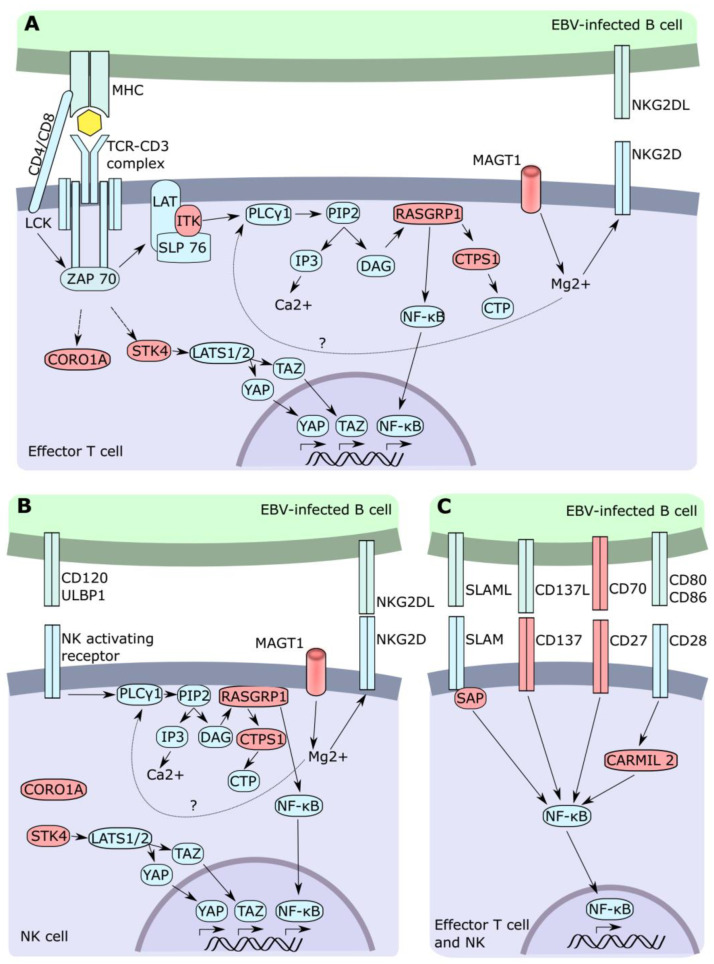
T cell and NK cell signaling following EBV-infected cell recognition. Cascade associated with TCR (**A**), NK activating receptor (**B**), and co-stimulatory (**C**) stimulation. Red color describes a gene with mutations associated with EBV.

Interestingly, while many IEI show a broad susceptibility to several pathogens including EBV, few have a restricted vulnerability to EBV only [17]. Mutations in genes involving non-redundant mechanisms of immunity against EBV lead to this EBV predisposition syndrome (Figure 1).

Although EBV has been associated with various malignancies [32,33,34], a healthy immune system is usually capable of controlling the infection. Most individuals remain asymptomatic, and EBV-associated cancer in immunocompetent individuals is relatively rare [35]. Disturbances of host immunity can tilt this balance to favor the virus, allowing its full oncogenic potential. Besides the persistent inflammatory environment caused by EBV viremia and the expression of oncogenic EBV proteins and nucleic acids, there is an inability to kill transformed cells due to defects in cytotoxicity in certain IEI [36]. In the IEI discussed below, the mechanisms involved in the immunity against EBV are dysfunctional leading to immune dysregulation and malignancies.

## 3. CD27-CD70 Deficiency

CD27 is a co-stimulatory receptor expressed constitutively in a variety of lymphocytes, such as NK and T cells [37]. It binds to CD70 resulting in nuclear factor kappa-light chain-enhancer of activated B cells (NF-kB) pathway activation [38]. CD70, on the other hand, is only transiently inducible upon stimulation on T, B, NK, and dendritic cells [39,40,41,42]. However, upon infection with EBV, CD70 is upregulated on B cells [43]. High levels of CD70 are also observed in B and T cell lymphoma and many solid tumors [44]. Murine models have shed light on the CD27–CD70 interaction. CD27 co-stimulation induces T cell development [45], increases CD8^+^ T cell activation [46,47,48] and cell survival, and further contributes to the differentiation of CD8^+^ T cells into memory cells [37,47,49].

Hypogammaglobulinemia clinical features of CD27- and CD70-deficient patients mainly result from EBV-associated immune dysregulation. They present with severe IM, lymphoproliferative disease (LPD), lymphoma, and hemophagocytic lymphohistiocytosis (HLH). In a retrospective study, nearly half (11/21) of CD70-deficient and 36% (12/33) of CD27-deficient patients developed lymphomas, with Hodgkin’s lymphoma (HL) being the most common malignancy (Table 1) [50,51]. The exact mechanisms involved in the defective immune response are still unknown. However, T cells from those patients show an altered phenotype, decreased EBV-specific expansion, and reduced cytotoxicity towards EBV-transformed B cells [43,50,52]. Additionally, CD27 and CD70 might play an important role in immune control of malignancies, even irrespective of EBV infection. In fact, the axis has the potential to induce expansion of effector T cells, break tolerance, and activate response in non-immunogenic tumors; several drugs—such as Varlilumab, SGN-CD70, SGN-7, and MDX-1203—targeting the CD27–CD70 axis are currently being tested in cancer therapy [44,53].

## 4. CD137 (TNFRSF9, 4-1BB) Deficiency

CD137 (also known as 4-1BB and TNFRSF9) shows many similarities with CD27. Both receptors are part of the TNFR superfamily and act as co-stimulatory receptors, increasing T cell proliferation, survival, cytokine production, and cytotoxicity. Unlike CD27, which is constitutively expressed by resting T cells, CD137 is induced after cell activation [47,108]. The expression of those receptors at different stages could explain why CD27 engagement favors the formation of effector T cells, while CD137 induces a more robust long-term immunity and secondary response [46]. CD137 ligand is expressed by dendritic cells, macrophages, and activated T and B cells, including EBV-infected B cells [47,108].

EBV-specific T cells from CD137-deficient patients presented lower interferon (IFN)-γ and perforin expression and showed impaired expansion in response to EBV-infected B cells compared to healthy cells. Similar results were also observed following CD137 blockage in T cells from healthy donors, highlighting its non-redundant role in the immune response against EBV [62,63]. Susceptibility to EBV was a common clinical feature among the patients described. Chronic EBV viremia. EBV-associated HLH and lymphoma were present in the majority of patients (Table 1) [61,62,63]. Interestingly, Rodriguez et al. suggested an incomplete clinical penetrance in one of two siblings described. Though both were carrying the same mutation in *TNFRSF9* and were EBV viremic, only one sibling developed symptoms. Importantly, specific CD8^+^ T cell responses towards LCL were impaired in both kindreds. The symptomatic sibling further showed digenic mutations in the *PIK3CD* gene (causative of activated PI3 kinase delta syndrome) that might have further contributed to the EBV-related clinical phenotype [61].

## 5. ITK Deficiency

Interleukin-2 inducible T cell kinase (ITK) is a member of the Tec family tyrosine kinases with a crucial role in mediating antigen receptor signaling in T cells. Following T cell receptor (TCR) engagement, the CD3 immunoreceptor tyrosine-based activation motifs (ITAMs) are phosphorylated by lymphocyte-specific protein tyrosine kinase (Lck). It allows zeta-chain-associated protein kinase 70 (Zap-70) to bind to phosphorylated ITAMs and subsequently to phosphorylate adapters of linker for activation of T cells (LAT) and the SH2 domain-containing leukocyte protein of 76kDa (SLP-76). ITK is recruited to the phosphorylated LAT/SLP-76 adapter complex, and together they activate phospholipase Cγ1 (PLCγ1). Activated PLCγ1 hydrolyzes phosphatidylinositol 4,5-bisphosphate (PIP2) to produce the second messenger molecules inositol 1,4,5-trisphosphate (IP3) and diacylglycerol (DAG). IP3 induces intracellular Ca^2+^ release, while DAG induces NF-κB and MAPK/ERK pathways [57,109].

ITK is not indispensable for TCR downstream signaling, it rather acts as an amplifier. Therefore, although some processes are barely affected, the development and differentiation of T cells might follow abnormal paths. ITK deficiency leads to a skewed Th1 response in detriment of the Th2 response, favors Treg differentiation over Th17, induces development of “innate like” CD8^+^ T cells, and abrogates development of NKT cells [54,110,111,112,113,114,115,116]. ITK-deficient CD8^+^ T cells show delayed effector function upon activation, decreased proliferation, and intrinsic defects in degranulation. Interestingly, those defects could be rescued by increasing costimulatory signals, such as prolonged IL-2 stimulation or the addition of IL-12 [117]. Intraperitoneal infection of ITK^−/−^ mice with murine gammaherpesvirus-68 (MHV-68) leads to latent intestinal infection, which develops into lethal colitis [118].

T cells from ITK-deficient patients were also reported to have low or delayed Ca^2+^ flux upon TCR stimulation with anti-CD3 [119]. Clinical features include hypogammaglobulinemia, EBV viremia, EBV-induced LPD, and lymphoma. Most commonly, HL has been observed in the reported patients (38%, 8/21). To date, no asymptomatic and/or EBV-naïve patient has been identified; therefore, it is still not clear whether the increased risk of developing lymphomas is also present in the absence of EBV. Nevertheless, the high incidence of HL, together with the fact that all HL and HL-like patients were EBV seropositive and expressing latency II proteins, suggest that ITK is involved in the immune control of EBV-associated oncogenesis [120].

## 6. RASGRP1 Deficiency

Similar to ITK, the nucleotide exchange factor RAS guanyl-releasing protein 1 (RASGRP1) is a secondary TCR messenger. Following increased DAG production by PLCγ1, RASGRP1 is recruited to the membrane and activates the small G protein RAS that in turn activates the cascade of MAP kinase (also known as Raf-MEK-ERK kinases) [121]. RASGRP1 is expressed on lymphocytes and its deficiency in NK and CD8^+^ T cells leads to defective proliferation and cytotoxic function [104,105]. Salzer et al. showed that although those cells had an increased expression of perforin and granzyme B, the release of cytotoxic granules was impaired [105]. However, this was not observed in other studies [104]. CD27/CD70-induced proliferation was also disturbed in RASGRP1-deficient T cells [104]. Given the importance of this pathway to control EBV-infected and transformed cells (discussed above), the same mechanism could lead to EBV susceptibility in RASGRP1-deficient patients. T cells from these individuals show a reduced cytidine triphosphate (CTP) synthase 1 (CTPS1) expression, an enzyme with a key role in DNA replication (discussed below). Deficiency of CTPS1 expression has been attributed to defective T cell proliferation in those individuals [104].

RASGRP1-deficient patients commonly show recurrent infections, inverted CD4^+^: CD8^+^ T cell ratio, poor T cell proliferation, defective NK cell function, autoimmunity, and EBV-associated lymphoma. Six out of nine patients developed EBV-associate malignancies, among them two were diagnosed with diffuse large B cell lymphoma (DLBCL), two with HL, one with low-grade lymphoma, and one with polymorphic B cell lymphoma [101,102,103,104,105].

## 7. CTPS1 Deficiency

CTPS1 is a key enzyme for de novo synthesis of CTP, a limiting nucleotide in cells, and therefore, essential for DNA replication. Resting T cells express rather low levels of CTPS1, but it is readily upregulated after TCR stimulation in accordance with its requirement for DNA synthesis and proliferation [98,122,123]. As expected, CTPS1-deficient cells exhibit impaired proliferation in response to TCR engagement (IL-2-induced proliferation remains unaffected), but other T cell functions, such as cytokine production and cytotoxicity, are not affected [98]. In light of the massive T cell proliferation required in EBV control [18,19], it is not surprising that CTPS1-deficient patients manifest with EBV-associated immune dysregulation. Common clinical features include severe IM and EBV-induced LPD, three out of 28 patients also developed EBV-driven lymphomas. Recurrent viral infections with other viruses, such as varicella-zoster virus (VZV) and human herpesvirus 6 (HHV6), were also common [96,97,98,99,100].

## 8. MAGT1 Deficiency (XMEN Syndrome)

Mutations in the gene encoding the magnesium transporter protein 1 (MAGT1) are causative of XMEN (X-linked immunodeficiency with magnesium defect, EBV infection, and neoplasia) syndrome. This disease was initially described in 2011 in two males showing recurrent pulmonary infections, low CD4^+^ T cells, and EBV-induced LPD and lymphoma [124]. Following TCR activation, T cells show a rapid Mg^2+^ influx that was abrogated in MAGT1 deficiency. It was thought that Mg^2+^ was a second intracellular messenger of the TCR linked with PLCγ1 activation and subsequently Ca^2+^ influx upon TCR activation [124,125,126]. Recently, Ravell et al. revealed another function of MAGT1, which could not yet be clearly attributed to intracellular Mg^2+^ transport. It was shown that defects in *MAGT1* cause glycosylation errors in specific subsets of glycoproteins, including NKG2D and CD70 expressed by immune cells [59]. NKG2D expressed in NK and CD8^+^ T cells plays a crucial role in killing EBV-infected and transformed cells, and its decreased expression makes it a perfect biomarker [127,128,129]. If poorly glycosylated, these receptors are prematurely degraded, leading to a low surface expression and subsequent disturbed effector function against EBV-infected targets [59,130]. Hence, besides recurrent infections, CD4^+^ T cell lymphopenia, hypogammaglobulinemia, and lymphadenopathy, the clinical phenotype of patients with MAGT1 deficiency includes high susceptibility to EBV-induced LPD and malignancies (14 of 37 reported patients). Again, HL was the most prevalent lymphoma (7 of 14 patients) (Table 1). Interestingly, EBV-naïve patients also frequently suffered from lymphadenopathy.

## 9. Coronin 1A Deficiency

Coronin 1A (coded by *CORO1A*) belongs to a family of coronins that is highly expressed in leukocytes. They are actin-binding proteins, which regulate cytoskeletal remodeling in response to extracellular signals. They modulate processes such as migration, phagocytosis, and cell polarization [131,132]. One of the most striking phenotypes of coronin 1A deficiency is the lack of naïve T cells. Interestingly, effector and memory T cell survival is barely affected and the intact thymus in these patients suggests normal T cell development [64,65,66,67,68]. It was initially believed that the accumulation of F-actin due to lack of coronin 1A activity and subsequent apoptosis was responsible for naïve T cell reduction [132]. Further studies did not confirm this but associated this finding with poor Ca^2+^ mobilization [133,134]. Finally, T cell lymphopenia was also thought to be a consequence of impaired thymic egress. Nevertheless, this hypothesis was based on defective egress observed in a murine model with a gain-of-function mutation (E26K) [64], while most patients have been harboring loss-of-function mutations in *CORO1A*.

Besides some individuals who manifest with a profound T cell reduction, i.e., complete SCID (severe combined immunodeficiency) phenotype, most coronin 1A-deficient patients reported to date suffered from recurrent (viral) infections, and an inability to control EBV, leading to EBV-associated LPD and lymphomas (Table 1). Although the mechanisms which make coronin 1A-deficient patients prone to EBV infection are still not clear, it is likely that a reduced EBV-specific CD8^+^ T cell expansion due to T cell lymphopenia plays a major role. Additionally, coronin 1A-deficient NK cells show diminished cytotoxicity and impaired degranulation caused by the accumulation of F-actin at the immunological synapse [135].

## 10. STK4 (MST1) Deficiency

Serine-threonine kinase 4 (STK4, also known as mammalian sterile 20-like 1, MST1), is a key kinase involved in the signaling of the canonical and non-canonical Hippo pathway. In the canonical path, STK4 phosphorylates large tumor suppressor kinases (LATS1/2), which further activate the yes-associated protein (YAP) and transcriptional co-activator PDZ-binding motif (TAZ). Activated YAP and TAZ act as transcription factors and induce the expression of various genes controlling cell growth, proliferation, and differentiation [136]. Through the non-canonical Hippo pathway, STK4 exerts a variety of other functions on immune cells, such as extravasation and vesicle trafficking of neutrophils [74,137], humoral immunity [77] and T cell migration, development, and function [76,138,139]. T cells from STK4-deficient patients show reduced proliferation upon stimulation [69,70,71,72,73,74,75,76,77,78,79,80]. Nehme et al. could link decreased T cell proliferation with elevated T cell apoptosis due to increased FAS expression on the T cell surface [78]. T cells from STK4-deficient patients also exhibit defective transwell migration in response to the chemokines CCL19, CCL20, and CXCL11, which is linked to lower expression of CCR7 and L-selectin in T cells [74,78].

STK4-deficient patients suffer from recurrent bacterial, fungal, and/or viral infections, including EBV-associated LPD, intermittent neutropenia, T and B cell lymphopenia, and increased risk of autoimmune diseases and lymphoma. Although around half of reported STK4-deficient patients have manifested with EBV-LPD and viremia, there is a further EBV independent risk of developing malignancies. Out of six lymphomas reported in five of the 28 patients, three were tested EBV-negative [79,80]. Several studies have associated *STK4* with tumorigenesis in mice [138]. Kim et al. showed that chromosomal instability present in STK4 knockout mice accelerated lymphoma development following mutagen treatment or p53 deletion [140]. Additionally, analysis of publicly available datasets of B, T, and NK cell lymphoma showed a significant decrease in STK4 expression in those malignancies [80]. Therefore, the lack of the antitumor capacities of STK4 should be considered as an additional risk factor in lymphoma development.

## 11. CARMIL2 (RLTPR) Deficiency

Capping protein regulator and myosin 1 linker 2 (CARMIL2, also known as RLTPR) is a protein expressed in many cell types, including lymphoid tissue and the gastrointestinal tract. It controls actin polymerization; hence it regulates a variety of functions as cell polarization and migration [141]. Despite its functions associated with actin, CARMIL2 acts as a messenger downstream of CD28, bridging the co-stimulatory receptor to the NF-kB pathway. CARMIL2-defective T cells demonstrate reduced proliferation, differentiation, and effector function following TCR-dependent CD28 co-stimulation [87,89]. The clinical features of CARMIL2 deficiency include recurrent and/or chronic bacterial, viral, and fungal infections, inflammatory bowel disease, and cutaneous manifestations. Patients present with low-level EBV-viremia. Interestingly, EBV-LPD or lymphoma has never been observed, instead 20% of the patients (8/44) developed EBV-associated smooth muscle tumors (SMT) [82,83,84,85,86,87,88,89,90,91,92,93,94,95]. The mechanisms are still unknown.

## 12. SH2D1A (XLP1 Syndrome) and XIAP Deficiency (XLP2 Syndrome)

X-linked lymphoproliferative disease type 1 (XLP1) is caused by mutations in *SH2D1A,* which encodes the signaling lymphocyte activation molecular (SLAM)-associated protein (SAP) [142,143,144]. SAP binds to the cytoplasmic domain of SLAM family receptors and regulates downstream intracellular signaling pathways following activation of SLAM receptors to their cognate ligands [145,146]. Engagement of the SLAM receptors 2B4 and NTB-A on SAP-sufficient CD8^+^ T and NK cells increases their cytotoxic effect. However, in SAP-deficient cells, stimulation of those receptors showed an inhibitory effect [147,148,149]. Furthermore, SAP signaling was only indispensable in response to B cells, as SAP-deficient CD8^+^ T cells were still able to kill other cell types, i.e., fibroblasts, monocytes, or dendritic cells [147,150]. This might explain why individuals with XLP1 do not show any susceptibility to other common viruses, such as cytomegalovirus (CMV), varicella-zoster virus (VZV), and human papillomavirus (HPV). While EBV shows a high tropism towards B cells, other viruses infect different cell types that are unaffected by the loss of SAP.

SAP^−/−^ mice infected with MHV-68 develop hypogammaglobulinemia and chronic inflammation with exacerbated proliferation of virus-specific CD8^+^ T cells and consequently increased tissue damage [151,152]. Similar symptoms were observed in XLP1 patients, who often manifest with severe EBV-induced IM and HLH, B cell lymphoma, and hypogammaglobulinemia. Surprisingly, although 25% of the cases of XLP1 develop B cell lymphoma, no significant difference between EBV-negative and EBV-positive individuals was observed. It is suggested that defects on NK and NKT cells, in addition to poor responsiveness of CD8^+^ T cells against B cells, play a pivotal role in the development of B cell lymphoma, rather than the ability of EBV to induce transformation [106].

In X-linked inhibitor of apoptosis protein (XIAP)-deficient patients, cytotoxicity of NK and CD8^+^ T cells are unaffected but CD8^+^ T cells lacking XIAP show increased apoptosis followed stimulation [153]. XIAP patients show high frequencies of EBV-related HLH; however, in contrast to SAP deficiency, inflammatory bowel disease manifestations are common, while B cell lymphomas are rare [154,155].

## 13. Conclusions

Since EBV was firstly identified 60 years ago, the immunological sequelae of EBV infection in immunocompetent and immunocompromised individuals has to a large extent been revealed [7,156]. Its association with malignancies, especially in numerous EBV-susceptible IEIs, is undisputed.

A shared characteristic observed in most IEIs susceptible to EBV is a CD8^+^ T cell dysfunction to various degrees (Figure 1). Although NK cells are also commonly affected, this feature was not observed in all genetic entities.

Furthermore, although the study of these IEIs contributed immensely to the knowledge of the interaction between the immune system and EBV, the exact mechanisms underlying lymphoma development are still not completely understood. Further studies will elucidate whether the high frequency of malignancies observed in some IEIs are linked to: (1) the uncontrolled EBV-infection, (2) the inability of the organism to control transformed cells independently of EBV, or most likely (3) a combination of both factors.

As the primary target of EBV is the B cell, it is not surprising that lymphoproliferative diseases in patients with IEIs are usually of B cell origin. However, our review did not include the rare but equally important as well as often fatal clinical manifestations of T/NK cell proliferative diseases. Fujiwara and Nakamura provide an in-depth review of the unique characteristics of chronic active EBV infection in IEIs with EBV-positive T/NK cell LPDs in a recent special issue of this journal [157].

The discovery of “new” IEIs have rapidly increased in the past years due to the increased application of next-generation sequences [158]. Novel discoveries should continue to rise as this technique becomes widely applied and new enhanced diagnoses are developed. As new cases arise, IEIs will remain a unique source of information to understand non-redundant pathways involved in the immunity against EBV and EBV-associated tumors. These studies will contribute to the development of better therapies, not only for individuals presenting those rare genetic diseases but also for more common diseases, such as severe IM, HLH, and cancer in immunocompetent people.

## Figures and Tables

**Table 1:** Inborn errors of immunity with high EBV susceptibility and disease.

Gene	Common Clinical Features	Number of Reported Cases	Types and Number of Malignancies in Reported IEI Patients
*CD27*	EBV viremia, LPD, HLH Recurrent infections Autoimmunity Lymphadenopathy Hypogammaglobulinemia	33 [50]	HL (9/33) [50] BL (1/33) [50] NHL (1/33) [50] DLBCL (3/33) [50]
*CD70*	EBV viremia, LPD, HLH Recurrent infections Autoimmunity Lymphadenopathy Hypogammaglobulinemia	21 [50,51]	HL (9/21) [50,51] BL (1/21) [50] NHL (1/21) [51]
*ITK*	EBV viremia, LPD Recurrent infections CD4^+^ T cell lymphopenia ↓ iNKT Hypogammaglobulinemia	21 [54,55,56,57,58]	HL (8/21) [56,57] HL-like (2/21) [57] DLBCL (2/21) [57,58] DLBCL-like (1/21) [57] BL (1/21) [57] NHL (1/21) [57] SMT (1/21) [57]
*MAGT1*	Chronic EBV viremia, LPD Recurrent infections ↓ NKG2D Inverted CD4^+^:CD8^+^ ratio Hypogammaglobulinemia Autoimmunity	37 [59,60]	HL (7/37) [59,60] BL (2/37) [59,60] Unclassified lymphoma (1/37) [59,60] Liposarcoma (1/37) [59,60] DLBCL (1/37) [59,60] EMZL (1/37) [59,60] Kaposi sarcoma (1/37) [59,60]
*TNFRSF9 (4-1BB/CD137)*	EBV viremia, LPD Recurrent infections Lymphadenopathy Hypogammaglobulinemia	8 [61,62,63]	HL (2/8) [62,63] DLBCL (1/8) [63] BL (1/8) [62]
*CORO1A*	EBV viremia, LPD Recurrent infections Lymphopenia	10 [64,65,66,67,68,69]	DLBCL (3/10) [67,68] Unclassified lymphoma (1/10) [65] Intracranial B cell lymphoma (1/10) [69]
*STK4*	EBV viremia, LPD Recurrent infections	29 [69,70,71,72,73,74,75,76,77,78,79,80]	HL (2/29) [78,80] DLBCL (1/29) [72] BL (1/29) [79] NHL (1/29) [80] PCTL (1/29) [81]
*CARMIL2*	EBV viremia, LPD Recurrent infections Inflammatory bowel disease	44 [82,83,84,85,86,87,88,89,90,91,92,93,94,95]	SMT (8/44) [84,85,86,89]
*CTPS1*	EBV viremia, LPD Recurrent infections Hypogammaglobulinemia	28 [96,97,98,99,100]	B-NHL (2/28) [98] CNSL (12/28) [100]
*RASGRP1*	EBV viremia, LPD Recurrent infections CD4^+^ T cell lymphopenia Lymphadenopathy Autoimmunity	9 [101,102,103,104,105]	DLBCL (2/9) [101] HL (2/9) [104] low grade unclassified lymphoma (1/9) [105] PBCL (1/9) [103] SMT (1/9) [104]
*SH2D1A* (XLP1)	EBV viremia, LPD, HLH Hypogammaglobulinemia	>100 [106,107]	Total lymphomas (25–30%) [106,107] DLBCL 30–40% [106,107] BL 40–60% [106,107] NHL 20–30% [106,107]

HL, Hodgkin’s lymphoma; BL, Burkitt’s lymphoma; DLBCL, diffuse large B-cell lymphoma; EMZL, extranodal subtype of marginal zone lymphoma; PBCL, polymorphic B-cell lymphoma; NHL, unclassified B cell non-Hodgkin lymphoma; CNSL, central nervous system lymphomas; SMT, smooth muscle tumor; PCTL, primary cardiac T cell lymphoma; LPD, lymphoproliferative disease; HLH, hemophagocytic lymphohistiocytosis.
